# Chicago Pathological Society, Stated Meeting, March 12

**Published:** 1888-04

**Authors:** 


### THE CHICAGO PATHOLOGICAL SOCIETY.

STATED MEETING, MARCH 12.

I. N. DANFORTH, M. D., President, in the Chair.

The session opened with a discussion of the paper, by Dr. J. B. Murphy, on "Gunshot Wounds of the Abdomen," which was read at the last meeting, and which appeared in the March number of the *Medical Journal and Examiner*.

Professor A. E. HOADLEY, in opening the discussion, said: The cases reported in the paper are of unusual interest, for they occurred in practically a new field in surgery. Until very recently laparotomy for gunshot wounds of the abdomen was not recognized as a justifiable operation, but now operations upon nearly all the abdominal organs have been demonstrated to be practicable. It is only within the last four or five years that operations upon the abdomen for the treatment of penetrating gunshot wounds have been practiced. The paper in itself, if there were no other evidence in favor of such operative interference, would seem to furnish facts sufficient to justify it.

The author of the paper reports four cases, in three of which there were wounds of the intestine in which there would have been, in time, more or less escape of fæcal matter into the peritoneal cavity, which would have set up fatal septic peritonitis. In none of those cases did peritonitis, as a complication, occur.

It is not my belief that it is necessary to pare the edges of small wounds where a bullet has simply traversed the intestinal wall, or that it is requisite to suture the mucous membrane. The mucous membrane of the intestine becomes ectropic; it almost completely occludes the wound so as to prevent, temporarily, the escape of fæcal matter. It is so redundant that suturing is unnecessary. If the peritoneal surfaces be brought together and sutured, that will be quite sufficient to seal the wound, and the mucous surfaces will take care of themselves.

In small wounds of the intestine, it will be sufficient to invert the mucous membrane, and, by continued suture, approximate the serous surfaces over the wound. These surfaces immediately become glued together, and the suture is soon buried in lymph. It will be found quite secure, and no doubt result in permanent and perfect union. The greatest objection to so many sutures, and such close coaptation of the wound, is the time required for the operation. Two hours were occupied in one of the cases reported, which is certainly a source of danger to the patient, who is already suffering from shock as a result of the injury. An ordinary bullet hole, made by a bullet of 32 or 38 calibre, through the intestine can be closed with four continuous catgut sutures, which are quite sufficient for all practical purposes. It can be done rapidly; it shortens the time, and consequently the patient is spared risk of additional shock.

Where blood clots exist of large size, whether from the mesentery or from the omentum, it is an easy matter to get them out of the meshes of the omentum and mesentery; but it happens sometimes in cases of gunshot wounds of the abdomen that there are a large number of blood clots, which vary in size from the head of a pin to that of the end of a person's finger. To get rid of them all would necessitate a long and tedious operation, and in doing it the peritoneum would probably thereby be wounded in many places. Large clots should be removed, but small ones should not be disturbed, because they will be absorbed without detriment to the case.

With reference to the irrigation of the peritoneal cavity, I would say that I consider a one or one-and-one-half per cent. solution of carbolic acid powerless to destroy the septic germs that may have found entrance into the peritoneal cavity from the intestinal canal; therefore it should be excluded. Boracic acid, while it is much more appropriate, is open to the same objection, that it is incapable of accomplishing the end for which it is used. We can accomplish practically nothing by the use of these disinfectants. The chief thing to be done is to wash out the peritoneal cavity with large and copious douches of warm (plain) water, sterilized, if practicable, and thus flood out what septic material may be present. We can use warm-water douches in these cases *ad libitum*. The water ought to be at a temperature of three or four degrees higher than the temperature of the body.

With regard to suturing the abdominal wound, while there is no practical objection to closing the wound with a double row of sutures, still it is open to criticism, for the reason that it takes up too much time and the results are no better. The more rapidly these abdominal operations can be performed the more successful will be the result. I am convinced that in all cases of perforating gunshot wounds of the abdomen it is the surgeon's duty to explore the abdominal cavity immediately, and not to wait for special symptoms to manifest themselves. In rare cases it might happen that the abdomen would be opened where the patient could have got well without it, but such cases are rare. In every case where the bullet can be traced in the direction of the abdominal cavity the abdomen should be opened early in order to find out what mischief has been done, and if it is opened under strict antiseptic precautions the patient's chances will probably not have been jeopardized by the operation.

I am in favor of opening the abdomen by incision in the median line, where it can be made available, for we can make a larger wound in that region with less shock to the patient than in any other, and have better command over the contents.

I have only one case to refer to in my own practice of treating gunshot wounds of the abdomen. The patient received a 32-calibre bullet about two inches to the left and one above the umbilicus. It passed through the abdominal cavity into the back, made three wounds in the intestine, traversed the omentum, and made two wounds in the mesentery. The wounds were closed with three or four continued superficial catgut sutures, including the peritoneal and muscular coats only. The mesenteric wound was not touched, as there was no bleeding at that point. The hole was half an inch in diameter, and I failed to see wherein we could improve the condition of things. If I had sutured it, I would have embarrassed some blood vessels on their way to the intestine. I thought it best to let it alone. The abdominal cavity was washed out with warm water. There was some escape of fæcal matter. There were no evidences of inflammation at the autopsy. The patient lived three and one-half days after the operation, and then died from continued shock. He had lost a large quantity of blood at the time of the injury. He had no pain after the operation; no tympanitis; no distressing symptoms; but continued to vomit at intervals up to the time of his death. Autopsy showed that the wounds were all perfectly closed and smooth; the stitches were all buried, and there was no inflammation in any portion of the peritoneal surfaces.

Dr. A. B. STRONG: The writer of the paper is to be congratulated on his success in saving two out of four cases operated on for gunshot perforation of the abdominal cavity. How best to close the wounded intestines is a matter of great practical importance, and one that surgeons are not at all fully agreed upon. It would seem that that manner of procedure by which the operator could most speedily and successfully close both the visceral wounds and abdominal cavity would be the preferable one. With prolonged exposure and manipulation one can hardly avoid increasing the amount of shock always present, and thus lessen the chances of success. It has been found that when the ragged edge of the wound is turned into the gut, and the serous surface with its subjacent muscular layer closed over it, the parts speedily and firmly unite. I do not see the necessity, then, of paring the wound and making two layers of sutures; neither do I see the advantage of closing the abdominal opening by uniting the peritoneum and other structures separately, when it can be done just as well, and at a saving of time, by the suture including at once all its layers. However, Dr. Murphy has had remarkable success, and it may be that the plan he has followed has advantages over the one that would seem to me to be the best. When we have unmistakable evidence that the ball has entered the abdominal cavity, I think, in the majority of cases, it is our duty to make an exploratory incision to ascertain exactly what damage has been done, and also to close the wounds in the simplest, safest, and most expeditious way at our command.

My experience in laparatomy for gunshot wounds of the intestine is limited to one case, where the ball entered on the left side about one and one-half inches above the anterior superior spine of the ilium, and passed downward into the pelvis. The case was not seen until twenty-four hours afterward; the patient was in collapse; the abdomen literally full of blood, which was oozing from the wound. The patient died before a large vein behind the peritoneum in the pelvis could be secured.

While speaking of these injuries, I wish to place on record a case of gunshot wound of the abdomen, in a girl fourteen years of age, that came under my observation twelve years ago. The ball, at short range, entered the abdomen two inches to the right of the median line and two inches below the navel. There was immediate paresis of the right leg, showing that the lumbar plexus had been injured. There was peritonitis. An expectant plan of treatment was followed and she ultimately recovered. With the surgical light of the present day this case would have been a proper one for laparotomy.

Professor Henry M. Lyman read a paper on "Alcoholism and Morphinism."

Just as the protracted use of alcohol finally produces chronic alcoholism, so the habitual consumption of morphine brings about a condition of chronic morphinism. Lancereaux, of Paris, has recently described the symptoms of this disease, which may be usually recognized about five or six months after the commencement of the habitual use of morphine. The victim finds, perhaps to his own surprise, that he can not do without the drug. Disorders of digestion soon appear; there is loss of appetite and of flesh; the countenance assumes a yellowish, earthy tint; the eyes grow dull, and only recover their vivacity after a fresh dose of the drug. The skin becomes dry; the muscles dwindle, and sometimes become œdematous. The patient now suffers with sleeplessness, or with distressing dreams and nightmare; there is loss of memory, and enfeeblement of the moral sense, though the intellectual faculties are still preserved. There are subjective pains experienced in all parts of the peripheral nervous system; sensations of pricking and of crawling, sufferings analogous to the osteoscopic pains of syphilis. General sensibility is also greatly modified—hyperæsthesia in certain regions with anæsthesia in others. Usually, there is an enfeeblement of the tendinous reflexes, though the plantar reflex is often exaggerated. The universal disturbance of nutrition is further illustrated by the rapid decay or loss of the teeth, and by the early development of baldness. The special senses are not specially injured, but sexual appetite is abolished; the female becomes sterile, and ceases to menstruate. According to Levinstein her entire uterine apparatus may become atrophied.

Between chronic alcoholism and chronic morphinism there are many points of resemblance; but Averbeck remarks that the alkaloid narcotics attack the nervous system first, and through its ruin destroy the general health, while alcohol first affects the organs of vegetative life, and through their destruction the nervous system is wrecked. In both forms of chronic intoxication, the frightful dreams, the disturbances of sensation, even the paroxysm of delirium, are quite similar. But in the digestive organs the symptoms of irritation and inflammation are more severe in the victim of alcohol. The morphine eater, furthermore, does not exhibit those fibrillary tremors of the lips that are so commonly observed in chronic alcoholism. The drunkard may grow fat, but the morphine victim manifests a repugnance for flesh food, and grows thin. Both diseases predispose the patient to pulmonary tuberculosis. A tendency to the formation of abscesses in the subcutaneous cellular tissue is usually encountered in chronic victims of morphinism, due to the enfeebled health of the patient, and to the fact that the drug is now so frequently introduced by the use of the hypodermic syringe.

Morphinism is most common among the higher classes. Of one hundred cases studied by Levinstein thirty-two were physicians, females, neurotic males, apothecaries, and hospital attendants; all whose daily occupation brings them in contact with sick persons and drugs are the most likely to fall into the habit of morphine-taking. Ludovic Jammes communicates to the Institute of France his observations in Cochin China and Cambodia, where he has seen cats, dogs, and monkeys intoxicating themselves with the smoke of their masters' pipes. These animals present a melancholy aspect, and they sleep much more than other healthy creatures. They seem to experience the same effects that are produced by opium upon the human species.

The experiments of Mme. Edwards and M. Pilliet, recently laid before the Biological Society of Paris, show that the daily hypodermic injection of morphine will produce, in animals thus treated, a fatty degeneration of the liver, with atrophy of the cortical cells, and development of granular masses along the border between the gray matter and the white substance of the cerebrum.

Dr. Henry Averbeck has recently contributed to the pages of the *Deutsche Medizinal Zeitung* a valuable article on the "acute neurasthenia" produced by sudden abstinence from the habitual use of morphine. He recognizes five grades of intensity in the toxic effects of the drug: (1) The period of excitement, which may be likened to the effect of good wine upon one who is not accustomed to its use; (2) the time of pleasure, so far as that can be obtained in a state of Nirvana-like repose, without joy or suffering; (3) the period of tolerance; now, the poison must be taken in continually increasing doses in order to procure any pleasure, or to ward off the terrible misery consequent upon partial abstinence; (4) symptoms of absolute poisoning; increasing doses avail nothing to produce pleasurable excitement; even the usual quantity of the drug causes nothing but suffering, with all the symptoms of chronic intoxication of the whole body; (5) death from the effects of the poison.

So profound is the prostration of the sufferer during the period of neurasthenia that his treatment should only be undertaken under the immediate presence and supervision of a physician. For this purpose a properly appointed hospital, or retreat, furnishes the best opportunity. Female nurses are preferable to male, on account of their greater gentleness, persuasiveness, and persistence in dealing with the sufferer.

About five hours after the last dose the storm of abstinence begins. This exhibits its greatest severity in those patients who have habitually consumed eight grains, or more, each day. The sufferer begs for his customary stimulant, weeps and howls if it is refused. He yawns and sneezes many times in succession; he is restless and full of pain. This period of distress continues from three to five days. At first the functions of the brain are thoroughly prostrated for several hours; then symptoms of spinal exhaustion appear. Coals of fire and streams of molten metal seem to ooze along the spine from the head to the sacrum. Respiration is hurried (100 times a minute); the pulse may beat 150 times a minute. During the third and fourth hours, the ganglionic nervous system begins to take part in the disturbance. The throat burns; the stomach feels as if wounded or scorched. Retching, vomiting, and diarrhœa follow. During all these hours the patient tries every form of movement in search of ease, but all in vain. Only during the utter exhaustion that succeeds to a fit of vomiting does he obtain momentary relief. After five to eight hours of such misery the storm subsides. The patient, in ninety-nine cases out of every hundred can only call for morphine. The phenomena of hysteria, of hypochondria, and of a general mental derangement will now appear. Personality seems lost; the majority of such subjects are now completely irresponsible. The patient is now in the stage of complete prostration. If he attempt to write, his hand will shake; letters and even whole words will be omitted, very much like what may be observed at the commencement of progressive paresis. The pupils are dilated, there may be double vision; the senses of smell and of hearing are abnormally exalted. Hyperæsthesia, rather than paræsthesia, is the characteristic condition during abstinence from morphine. The muscular apparatus is utterly powerless; the patient feels best in the horizontal position. The secretory organs are now in a state of great activity; only the sexual organs remain impotent. The urine frequently contains albumen or, possibly, sugar. As a consequence of the irritable weakness of every part of the body, every impression upon the sensory structure of the entire organization gives rise to a sensation of pain. This tremendous enfeeblement of the nervous system brings the patient to the verge of dangerous, of fatal, collapse. To avert this, alcoholic stimulants, especially champagne, are most useful. A return to small doses of morphine, hypodermically given, may become imperatively necessary. The danger of collapse is most to be feared from the third to the sixth day of abstinence. It is most likely to occur in cases of fatty degeneration of the heart, induced by the conjoined abuse of alcohol and morphine.

After a succession of paroxysms, like that above described, at the end of the fourth or fifth day, the first indications of convalescence appear. Appetite for food begins to revive. The greatest dangers and sufferings are past; but the patient is sleepless, enfeebled, and depressed in spirit. The nervous system is weak and irritable.

The prognosis is good so far as recovery from acute neurasthenia is concerned, but the dangers of relapse into the opium-habit are very great. Among physicians who have contracted the habit relapses occur in 90 per centum, among officers, in 75 per centum, of the cases.

The morphine habit can only be cured by total abandonment of the drug. The use of cocaine in its place is a great mistake. The only choice lies between two methods: Shall we abruptly terminate the use of morphine, or shall we proceed by gradual diminution of the daily dose? It seems more physiological, in dealing with a habit that has grown out of gradually increasing doses of the drug, to escape from its grasp by a gradual reversal of the process. It also seems so much more merciful and humane to remove the dog's tail inch by inch each day, in hopes that the good creature may thus become tolerant of the pain. The best way consists in the adoption of a modification of the method by immediate abstinence. The patient who has been accustomed to the daily consumption of fifteen grains must be reduced to a tenth of this quantity, and this tenth must be divided into three doses at intervals of eight hours. After the eighth day the amount should be reduced to a hundredth part of the original dose. The phenomena of acute neurasthenia will now appear. If collapse is threatened, small doses, varying from a fortieth to a tenth of the original, may be administered once or twice during the twenty-four hours, especially at bedtime, until the worst five or six days are spent. Good wine, champagne by preference, must be given freely. Levinstein's method of giving chloral hydrate in half-drachm doses, with about a quarter of a grain of morphine, is not advisable; it acts like a regular brain-poison. During the period of acute neurasthenia warm baths, of ten or fifteen minutes duration, should be given every morning and evening. These may be advantageously terminated by thoroughly showering the spine with water a little cooler than the bath. As soon as the appetite begins to revive, massage should be employed, and the baths may be made somewhat cooler.

The method of treatment above sketched is only applicable to ordinarily healthy patients who have contracted the opium habit. It would be in the last degree inhuman to undertake a cure with the victims of incurable and painful diseases. For such, especially in malignant or tubercular cases, and in hæmorrhagic uterine cancer, morphine affords the best means of relief and prolongation of life.

We have been told that in China the use of opium is no more injurious than the moderate use of good wine or beer among the better classes in Occidental countries. Dr. Edwards, the physician in charge of the Opium Refuge connected with the China Inland Mission, states that "in the villages a large proportion of the inhabitants are addicted to the habit—according to the estimation of the people themselves, at least 80 or 90 per centum of the men above twenty; 50 or 60 per centum of the women; many of the young people in their teens, and even some of the children. The common opinion here is, that if a man begins to smoke opium and smokes regularly for a month, he will at the end of that time have a craving which nothing but a further supply of opium will satisfy. At the end of a year he finds his craving so great that without his pipe he can not possibly do his work. What his craving is, only those who smoke opium know. The replies of those who have been questioned on this point generally resolve themselves into this: When the craving comes on, I feel bad all over; my bones and joints ache, and the pain makes me perspire very freely. I am so weak that I can not work, and I feel if I do not take another pipe I shall soon die."

Kiernan, of Chicago, and Hughes, of St. Louis, recommend the use of three-drachm injections of tincture of capsicum into the rectum as an antidotal stimulant in cases of opium-poisoning. The effect is prompt, but its use is sometimes attended with the disagreeable consequence of a severe proctitis for a considerable time afterward.

Professor LYMAN closed by saying: Of all the people spoken to on the subject not one has been heard to say a word in defense of it, but almost without exception they give it an unqualified condemnation. The smokers themselves are very often ashamed to confess that they are addicted to the habit, and especially is this the case among those who come as patients. In the face of these facts one wonders what experience a man can have had who says that *he can testify that opium-smoking is of itself absolutely harmless.*

The PRESIDENT asked Professor Lyman whether he had observed any particular effects from the use of the fluid extract of *avena*, to which Professor Lyman replied that he had been unable to satisfy himself of its value, and thought it was one of those drugs with an imaginary reputation.

The PRESIDENT: It is not to any extent an antidote for the effects of opium, is it?

Professor LYMAN replied that it is not.

### FIBROID TUMOR OF THE UTERUS.

The PRESIDENT exibited specimens of a fibroid tumor of the uterus, and made the following remarks: These specimens are from a uterine fibroid of 25 years' standing, in a lady 65 years of age. The history of the case, as near as it can be ascertained, is that about 25 years ago she first discovered the fact that she had a slight abdominal enlargement. She was then examined by two or three physicians in this city, and the growth was diagnosticated as "uterine fibroid, ovarian fibroma, sarcoma, cystic degeneration," etc. At one time—15 years ago—preparations were made for an operation, and the operators assembled, but for some reason or other it was abandoned. She then left this city for St. Paul, was there several times examined, but I have been unable to find out what diagnosis was made. She returned to Chicago about a year ago, and has been under my care since that time. I did not attempt to make a diagnosis, although I believed it to be a fibroid tumor. I did not propose to do anything for it, although the patient was in a good condition. But she was possessed with an almost insane desire to get rid of the tumor. It gave her so little inconvenience that I declined to operate. I did not call in counsel in the case. About six months afterward she went east on a visit, and was well enough, it seems, to climb the steps of the Capitol dome at Washington. From Washington she went to Brooklyn and New York, and kept up her excursions. She wrote back saying she had not felt so well for years. She was gone about two or three months, when she returned to the city feeling remarkably well; but two or three days after her return she began to have hæmorrhage from the uterus. She had little or no pain. I was called, and prescribed ergot in full doses, which arrested the hæmorrhage, and two or three days afterward these masses began to come away under the ergotic stimulation of the womb. They came away almost spontaneously, followed shortly after by the appearance of a very copious sanious discharge, which was offensive, and the patient's condition appeared to be alarming. I thought it possible that some of the remnants of the tumor might have been arrested in the mouth of the womb and might be drawn away by forceps. I introduced a Sims speculum and drew away a large mass of the tumor, but that produced no particular relief; the discharge continued to be copious and offensive. Seeing the condition in which the patient was, I had her etherized, and with placenta-forceps I drew away portion after portion of the tumor which appeared to be lying loose in the uterus and easily detached, until I had drawn away three times as much as you have here seen, and in a very offensive condition. I did not use a curette. Shortly after these masses were removed her pulse began to fail, and prudence dictated a suspension of operations. She came out of the operation without shock or exhaustion. Two or three weeks later this same sanious discharge appeared, and she returned for the removal of the rest of the tumor, or, as she expressed it, "wanted to be delivered." I again had her etherized, and removed all the detached portions of the tumor I could reach with a Thomas curette. The tumor appeared to be intramural, and to be a lobulated, fibrous mass as near as I could determine.

At the last operation, which was about two weeks ago, I removed what I believed to be all of the remaining portions of the growth and left only a denuded surface, which surface I denuded still more by the use of a curette, without much loss of blood or any great degree of exhaustion. After giving ergot, I ceased operations. The patient is improving from day to day, feels much better than she has for a long time, the offensive discharge has entirely ceased, and in every way the results of the case are very promising indeed.

Professor HOADLEY: Did the patient have the characteristic labor pains during the expulsion of the first mass of the tumor?

The PRESIDENT: Yes; she did.

Professor HOADLEY: Did the uterus contract after removing the mass?

The PRESIDENT: It contracted a little; it is day by day lessening its bulk, and I am still giving the ergot.

Professor HOLMES: What relation, if any, has this tumor to those forms that are apt to be melanotic?

The PRESIDENT: No direct relation. Melanotic masses may be found sometimes in fibroid tumors, but they are isolated.

Professor LYMAN: What was the condition of the os?

The PRESIDENT: It was the size of a quarter of a dollar, and then I dilated it with a Thomas dilator.

Dr. STRONG asked whether sarcomatous tumors of the uterus, as a rule, were very rapid in their growth?

The PRESIDENT: Not very rapid. The rapidity of a uterine tumor depends largely upon the time it occurs. If the menopause had not occurred soon after this tumor developed, it would probably have become malignant. It is exceedingly interesting to notice how long the tumor had been there, and yet remained benign.

Professor HOADLEY: The malignancy of a sarcoma depends upon the patient's power to resist it. Some sarcoma become rapidly malignant. In Dr. Danforth's case the tumor occurred just at the right time to favor its physiological resistance. The blood-supply was cut off by the menopause; that is the only way to account for its benignity.

